# Prioritized Research for the Prevention, Treatment, and Reversal of Chronic Disease: Recommendations From the Lifestyle Medicine Research Summit

**DOI:** 10.3389/fmed.2020.585744

**Published:** 2020-12-22

**Authors:** Yoram Vodovotz, Neal Barnard, Frank B. Hu, John Jakicic, Liana Lianov, David Loveland, Daniel Buysse, Eva Szigethy, Toren Finkel, Gwendolyn Sowa, Paul Verschure, Kim Williams, Eduardo Sanchez, Wayne Dysinger, Victoria Maizes, Caesar Junker, Edward Phillips, David Katz, Stacey Drant, Richard J. Jackson, Leonardo Trasande, Steven Woolf, Marcel Salive, Jeannette South-Paul, Sarah L. States, Loren Roth, Gary Fraser, Ron Stout, Michael D. Parkinson

**Affiliations:** ^1^Department of Surgery, University of Pittsburgh, Pittsburgh, PA, United States; ^2^Department of Medicine, George Washington University School of Medicine, Washington, DC, United States; ^3^Department of Nutrition, Harvard T.H. Chan School of Public Health, Boston, MA, United States; ^4^School of Education, University of Pittsburgh, Pittsburgh, PA, United States; ^5^American College of Lifestyle Medicine, Chesterfield, MO, United States; ^6^Community Care, Pittsburgh, PA, United States; ^7^Department of Psychiatry, University of Pittsburgh, Pittsburgh, PA, United States; ^8^Department of Medicine, University of Pittsburgh, Pittsburgh, PA, United States; ^9^Department of Physical Medicine and Rehabilitation, University of Pittsburgh, Pittsburgh, PA, United States; ^10^Institute for Bioengineering of Catalunya, Barcelona Institute of Science and Technology, Catalan Institute of Advanced Studies, Barcelona, Spain; ^11^Department of Internal Medicine, Rush University Medical Center, Chicago, IL, United States; ^12^American Heart Association, Dallas, TX, United States; ^13^Lifestyle Medical, Riverside, CA, United States; ^14^Department of Internal Medicine, University of Arizona, Tucson, AZ, United States; ^15^United States Air Force, Washington, DC, United States; ^16^Department of Physical Medicine and Rehabilitation, Veterans Administration Boston Healthcare System, Boston, MA, United States; ^17^True Health Initiative, Derby, CT, United States; ^18^Department of Pediatrics, University of Pittsburgh, Pittsburgh, PA, United States; ^19^Department of Environmental Health Sciences, University of California, Los Angeles, Los Angeles, CA, United States; ^20^Department of Pediatrics and Environmental Medicine, New York University, New York, NY, United States; ^21^Department of Family Medicine and Population Health, Virginia Commonwealth University, Richmond, VA, United States; ^22^Division of Geriatrics and Clinical Gerontology, National Institute on Aging, Bethesda, MD, United States; ^23^Department of Family Medicine, University of Pittsburgh, Pittsburgh, PA, United States; ^24^Phipps Conservatory and Botanical Gardens, Pittsburgh, PA, United States; ^25^Department of Medicine, Preventive Medicine and Public Health, Loma Linda University, Loma Linda, CA, United States; ^26^Ardmore Institute of Health, Ardmore, OK, United States; ^27^University of Pittsburgh Medical Center Health Plan/WorkPartners, Pittsburgh, PA, United States

**Keywords:** lifestyle medicine, chronic disease, inflammation, epigenetics, research methodologies, *in silico* modeling, nutrition, physical activity

## Abstract

Declining life expectancy and increasing all-cause mortality in the United States have been associated with unhealthy behaviors, socioecological factors, and preventable disease. A growing body of basic science, clinical research, and population health evidence points to the benefits of healthy behaviors, environments and policies to maintain health and prevent, treat, and reverse the root causes of common chronic diseases. Similarly, innovations in research methodologies, standards of evidence, emergence of unique study cohorts, and breakthroughs in data analytics and modeling create new possibilities for producing biomedical knowledge and clinical translation. To understand these advances and inform future directions research, The Lifestyle Medicine Research Summit was convened at the University of Pittsburgh on December 4–5, 2019. The Summit's goal was to review current status and define research priorities in the six core areas of lifestyle medicine: plant-predominant nutrition, physical activity, sleep, stress, addictive behaviors, and positive psychology/social connection. Forty invited subject matter experts (1) reviewed existing knowledge and gaps relating lifestyle behaviors to common chronic diseases, such as cardiovascular disease, diabetes, many cancers, inflammatory- and immune-related disorders and other conditions; and (2) discussed the potential for applying cutting-edge molecular, cellular, epigenetic and emerging science knowledge and computational methodologies, research designs, and study cohorts to accelerate clinical applications across all six domains of lifestyle medicine. Notably, federal health agencies, such as the Department of Defense and Veterans Administration have begun to adopt “whole-person health and performance” models that address these lifestyle and environmental root causes of chronic disease and associated morbidity, mortality, and cost. Recommendations strongly support leveraging emerging research methodologies, systems biology, and computational modeling in order to accelerate effective clinical and population solutions to improve health and reduce societal costs. New and alternative hierarchies of evidence are also be needed in order to assess the quality of evidence and develop evidence-based guidelines on lifestyle medicine. Children and underserved populations were identified as prioritized groups to study. The COVID-19 pandemic, which disproportionately impacts people with chronic diseases that are amenable to effective lifestyle medicine interventions, makes the Summit's findings and recommendations for future research particularly timely and relevant.

## Introduction

A recent 60-years review of mortality in the United States, with special focus on the past two decades, revealed declining life expectancy that is particularly pronounced in ages 25–44, typically Americans' most productive years. The primary causes of this increase in midlife mortality were unhealthy behaviors, such as drug and alcohol use, suicides, hypertensive diseases, diabetes, and obesity which collectively have taken a (largely preventable) toll on both the length and quality of life of Americans relative to peers in other high-income countries (1).

The disparity between leading causes of death and federal research spending was recently highlighted in a review of the U.S National Institutes of Health (NIH) Prevention Research Portfolio for the period 2012–2017 (2). Eleven-thousand studies characterized as primary or secondary research studies, comprising 17% of total NIH research spending, were identified for analysis. Only 26% of those studies addressed a leading cause of death as the outcome of interest (e.g., heart disease, cancer, injury, etc.). One third of the studies addressed one of the leading risk factors for death, such as diet, tobacco use, high blood pressure, weight, elevated blood sugar, and high cholesterol. A third addressed the leading causes of disability. Randomized, placebo-controlled interventional trials, the highest level of evidence for clinical interventions, comprised 25% of spending. Only 3% of the studies addressed multiple risk factors or behaviors (as they typically co-occur in a population); 20% involved youth; 10% studied elderly, urban populations or women, particularly pregnant women; 5% or fewer of the studies focused on low income, rural, sexual and gender, underrepresented race/ethnicity groups, institutionalized, or the disabled populations. Essentially no studies evaluated the use of lifestyle interventions to treat and reverse the major chronic diseases that increasingly are the primary root causes of premature mortality and costly morbidity. This disparity is especially glaring given the major advances across a host of basic and translational science methodologies that have received extensive NIH funding and have helped set the stage for Precision Medicine application across a wide range of diseases.

Lifestyle Medicine addresses the use of a whole-food, plant-predominant diet, regular physical activity, restorative sleep, stress management, avoidance of risky substances and positive emotions/social connection as a primary therapeutic modality for treatment and reversal of chronic disease (3). Given the aforementioned paucity of studies focused on lifestyle medicine and the rapid methodological advances impacting numerous aspects of the biomedical enterprise, leading national experts were invited to review existing knowledge and gaps relating lifestyle behaviors to common chronic diseases, such as cardiovascular disease, diabetes, cancer, inflammatory- and immune-related disorders and other conditions. The 2-days Lifestyle Medicine Research Summit (4) prioritized major research questions from basic science to population health. This intentionally broad but interrelated perspective included lifestyle-induced or -associated inflammation; immune dysfunction; cellular dysbiosis; microbiome alterations; neuroplasticity; genomics, epigenetics, proteomics, metabolomics, and computational and systems approaches for assessing health and disease. Innovative research methodologies considered included new approaches to clinical trials design, *in silico* (computer) modeling, and opportunities for population-based and/or banked serological/specimen investigations using existing cohorts. The foundational and synergistic roles of sociocultural influences, built environment, and environmental exposures as well as the need to focus on underserved and understudied populations were also emphasized. Notably, the Summit was the first lifestyle medicine meeting with so wide-ranging an interdisciplinary scope.

**Figure 1:**
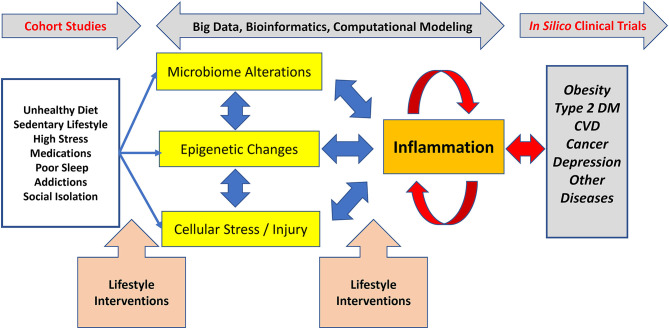
Lifestyle-associated pathogenesis, interventions, and emerging methods for lifestyle medicine research. Unhealthy lifestyles cause dysregulation in the microbiome, epigenetic changes, and various types of cellular stress and injury which, together, drive inflammation. In turn, inflammation can drive further derangements in the microbiome, can cause distinct epigenetic changes, and can drive further cellular stress and injury. This positive feedback leads to a process wherein inflammation becomes chronic and self-sustaining, ultimately resulting in chronic diseases, such as Type 2 diabetes. The non-linear nature of these processes means that simple reductionist approaches to understanding the connections between lifestyle mismanagement and chronic disease are likely to fail. Effective lifestyle interventions prevent, treat, and reverse common chronic diseases. To accelerate the adoption and dissemination of clinical lifestyle medicine interventions, there is a need for cutting edge biological and computational approaches decipher this complexity. Adapted from Figure 3, Bodai et al. (5) with permission from The Permanente Press.

The potential clinical impact of applying lifestyle medicine practices to the leading causes of disease begins by understanding their biological impact, especially on the common, inflammation-mediated pathways leading to multi-organ and -system disease (5, 6) [Figure 1; adapted from Figure 3, Bodai et al. (5) with permission from The Permanente Press]. The health effects of diet, sedentary lifestyle, chronic stress, medications (often used to treat lifestyle-determined conditions), and other aspects of lifestyle (sleep, substance use, emotions and attitudes, positive psychology and social connection) are mediated through common subcellular, epigenetic, and other mechanisms and to create dysbiosis (a state in which gastrointestinal flora become unbalanced or perturbed), cellular stress and injury (often in the form of oxidative stress). These biological pathways often culminate in the inflammatory response, which then feeds back to drive further cellular stress, dysbiosis, and related epigenetic changes to create a self-sustaining state of chronic inflammation. In turn, chronic systemic inflammation is an early precursor for heart disease; Type 1 and 2 diabetes and depression; and a variety of other endocrine, autoimmune, rheumatologic, and neurological disorders (Figure 1). A multitude of computational and systems biology approaches are elucidating the interconnections among these processes in the context of specific diseases and distinct sub-populations. While this emerging knowledge has been helpful in elucidating biological mechanisms, it has not made a major impact on clinical practice or lifestyle medicine research.

The Summit convened cross-disciplinary subject matter experts to address these knowledge gaps by exploring the synergy among emerging sciences, innovative research methodologies and populations, and the major domains of lifestyle medicine. We hypothesized that this multi-dimensional and multi-disciplinary intersection of perspectives (Figure 1) has the potential to create new insights and cross-sectoral strategies to articulate and explore potential breakthrough research priorities for scientists, clinicians, and funding agencies who are seeking more effective means to address health, medical care, and population health challenges. The overarching goal of the Summit was to inform the research community as well as public and private funding agencies. It sought to “close the gap” between what is known and what needs to be discovered to accelerate the further development and deployment of lifestyle medicine practices primarily but not exclusively in the clinical setting.

## Foundational Understanding of Common Pathways: Inflammation, Epigenomics, the Microbiome, and Neuroplasticity

Inflammation, a prototypical complex system with positive and negative feedbacks (7), plays a key role in linking lifestyle (mis)management to downstream pathobiology (8) (Figure 1). Inflammation is an adaptive response to stress that regulates multiple physiological processes, including the immune response, both positively and negatively (8). In turn, inflammation is itself regulated at multiple levels, including a newly appreciated role with metabolites such as glucose and lactate (9) and for related epigenetic changes (10) that are affected by lifestyle (e.g. diet) (11) (Figure 1). Furthermore, inflammation regulates – and is regulated by – changes in the microbiome (Figure 1) and, ultimately, by neural circuits that also regulate the microbiome and *vice versa* (12). This “neuroplasticity,” or the changeability of brain processes and pathways formerly presumed to be “fixed,” which is increasingly understood as a significant component of the “mind-body” mechanism (13). Microbiome changes occur with diet, sleep, exercise, stress, addiction, and the built as well as natural environment (14).

The conceptual framework for aging have also changed, from focusing only at the terminal stages of life to thinking across the entire lifespan, including the construct of “healthspan” (i.e., the length of time that the person is healthy, not merely alive) (15). Understanding how the body brings itself into balance (homeostasis) and the beneficial effects of stress below a certain level (hormesis) are also areas of increasing research interest related to healthspan. How much one exercises (type, frequency, duration, timing, intensity, etc.) and how much (and what) one eats (pattern, timing, quality, etc.) are clearly linked to potentially persistent epigenetic changes (11). We expand on these concepts below.

Inflammation, a common end pathway for multiple diseases promoted by lifestyle behaviors and “mismanagement,” is often assessed using relatively non-specific biomarkers, such as C-reactive protein and the erythrocyte sedimentation rate. When studying the impact of lifestyle on specific inflammatory mediators it is important to note that correlation does not imply causality. Accordingly, there is a major need to combine targeted inflammation biomarkers (e.g., interleukin-6 or tumor necrosis factor-α) with an unbiased approach involving systems and computational biology that takes into consideration state-of-the-art ‘omics methodology and concepts in order to define the impact of lifestyle (mis)management on inflammation-associated chronic diseases (Figure 1). As discussed in the following sections, this need introduces the intertwined discussions of emerging computational methodology and appropriate study cohorts.

## Current and Future Research Models: Roles for Big Data, Artificial Intelligence, and *in silico* Modeling

In parallel with the growing application of computational methods to biomedicine in general, machine learning (the method by which computers interpret data, also lumped together with “Big Data” (which refers to the increasingly large amount of data of multiple types obtained from multiple sources that are the starting point for machine learning) or “Artificial Intelligence” (involving a computer's ability to make decisions based on data analysis that would otherwise require human intervention) (16) have found increasing use in lifestyle medicine, predominantly in the area of epidemiology (Figure 1; see below). Collectively, these approaches are based on the use of computer algorithms to generate statistically grounded models based on various correlations within data, with the goal of discovering hidden or non-intuitive (and often non-linear) associations or predictive features; there has been much discussion of the application of machine learning approaches in medicine, though this is still an emerging field (17). Epidemiology is advancing in many directions, building upon a longstanding track record of successful application of traditional epidemiological methods. The past 10–15 years have brought a major paradigm shift known as “Systems Epidemiology,” based on nutritional genomics, nutritional metabolomics, the nutritional microbiome, and metagenomic analysis (12, 18). These “omics”-based methods have been facilitated by the rapid improvements in technology and concomitant drop in cost, especially for genomic sequencing. The Big Data approach has also improved dramatically in the past 10–20 years, with initial primary focus on traditional risk factor analysis now expanded to high-dimensional data analysis, network analysis, and pathway analysis (12, 18). One key pitfall to these statistically based approaches is that many identified associations do not necessarily reflect causal relationships, though well-performed studies address key confounders and incorporate biological pathways. Another, related pitfall to these approaches, the so-called “curse of dimensionality,” is that in a very real sense the data generated are actually “Small Data,” wherein very large amounts of data (e.g., 400,000 DNA methylation sites) are generated from a relatively small number of subjects (e.g., 400 patients, often sampled at only one or two time points), which can lead to highly over-fit statistical models without external validation (19). Thus, it is difficult to try to use purely data-centered approaches to get a comprehensive understanding of the dynamic impact of lifestyle on biology and *vice versa*. For example, network diagrams are static representations of data that can help suggest connections and formulate hypotheses (including in highly complex settings, such as the brain connectome or acute inflammation). However, even when derived from data obtained at multiple time points, these static models cannot be played backward to understand where the changes came from or to discover emergent phenomena (20). An alternative approach, “mechanistic computational modeling,” involves encoding key interactions and mechanisms as differential equations or “agents” in agent-based models. Mechanistic modeling is dynamic modeling, meaning that the models are meant to be played forward and tested against data obtained over time, and can also be played backward to gain insights as to why a given phenomenon might have occurred. This modeling approach can be used to generate “digital twins” (by calibrating these mechanistic models to data in individuals) and virtual population—or virtual (*in silico*) clinical trials. Notably, these methods were pioneered in studying inflammation (20). Importantly, data-driven and mechanistic modeling approaches can and should be used in tandem to leverage their respective strengths while minimizing their weaknesses (20).

Lifestyle medicine would be a key domain wherein *in silico* clinical trials and other forms of “Network Medicine” could prove invaluable (Figure 1), since it is often neither possible to carry out true randomized controlled trials of dietary or other lifestyle modifications (21) nor to reduce symptoms to single underlying mechanistic causes (20) (see also below). The use of computational models to capture the multi-factor and multi-scale organization of pathologies introduces a paradigm in which theory-driven biology can lead to theory-driven, rather than largely empirical, clinical interventions (20). Examples include work in stroke rehabilitation, where neuroscience theory has been used to develop technology-driven, patient-centered solutions shifting interventions from peripheral manipulation to central functional re-organization using AI techniques combined with virtual reality content delivery (22). Ultimately, however, computational models are by definition hypotheses (20). Both mechanistic models and some classes of machine learning models (e.g., a network depiction of the data), are forms of hypotheses about relationships, and so there should not be a tension between these *in silico* approaches and the “pure” hypothesis-driven approaches (23, 24).

The foregoing discussion emphasizes the crucial need to define the populations that should be studied and the types of data to be collected to facilitate *in silico* approaches. Populations exist within communities, defined either geographically or by shared demographic, cultural, behavioral or disease characteristics. The data typically used in Systems Epidemiology analyses are derived from the subjects of cohort studies, observational cohorts, and dietary intervention studies, as well as from electronic health records (25). For example, these include large and well-established cohorts, such as the Framingham Heart Study (26), the Nurses' Health Study (27), the UK Biobank (28), etc. In the past several decades, these studies have collected extensive data on diet, lifestyle, genetics, biomarkers, and health outcomes, which now also incorporates technology-derived elements, such as geographic information systems (GIS) data and other “digital phenotyping” data using multiple personal devices. The Adventist Health Studies conducted by Loma Linda University have involved nearly 100,000 participants for more than six decades (29). This long-term epidemiologic research has assessed the long-term relationship between lifestyle, diet, disease, and mortality among Seventh-day Adventists. The Department of Defense (DoD) Serum Repository, in existence since 1985, collects 2 million serum specimens per year from active duty military members for surveillance of operationally-relevant conditions (30). The DoD also conducts the Millennium Cohort Study, initiated in 1999 to understand military service members' health longitudinally both during and after a military career (31). Innovative, birth-to-high school age, community-based cohorts, such as the newly launched Pittsburgh Study (32) create new partnerships and opportunities to understand the complex interactions among individual, family, community, and environmental factors that contribute to disease, educational and social outcomes that are amenable to evidence-based interventions. The NIH “All of Us” Cohort study creates another unique opportunity to combine “Big Data” analytics and with state-of-the-art *in silico* modeling capabilities to examine the complex interactions between genomics and the environment on health and disease outcomes (33).

A major focus of any lifestyle medicine study—whether observational or interventional—is data quality. This is particularly important because most lifestyle data are self-reported. Keys to improving the quality of self-reported data include the use of validated and standardized questionnaires, the repeated measures of diet and lifestyle, high follow-up rates, and the complementary use of objective biomarkers. When the field moves into the “omics” era, although the genotyping data are highly accurate, the “noise” existing in other types of “omics” data (especially epigenomics and meta-genomics data) are substantial, and thus quality control and careful data analyses and interpretation are critical. An important element in understanding the biologic impact of lifestyle is the dynamics of molecular and cellular processes, which dovetails with the use of dynamic computational modeling methods (Figure 1). Intertwined with the need to obtain data at multiple time points is the need for retention of subjects in the study cohort for years or decades, which is a major challenge. Another major challenge is the use of appropriate methodology to synthesize the vast amount of data through systematic reviews and meta-analyses, which should be conducted with caution and interpreted in light of the broader context of the field (25).

Patients exist within communities, embedded in complex interactions with their environment—the social, cultural, political, pharmacological, and economic environment—which is intrinsically connected to their lifestyle and their health. This complex collection and integration of multiple personal and potentially sensitive data sources will require intensive efforts, and new approaches to ensure the understanding, trust, and consent of studied populations. This may be particularly challenging when partnering with groups and communities that have been underserved or understudied and discriminated against, and/or are often the most susceptible to poor health outcomes associated with lifestyle behaviors and unhealthy environments.

## Lifestyle Medicine Domain-Specific Findings and Recommendations

Subject matter experts for each of the lifestyle medicine domains provided an overview of the current scientific knowledge and made recommendations for prioritized research to accelerate the understanding and use of clinical application to prevent, treat and reverse those diseases.

### Nutrition Overview and Prioritized Research

#### Cardiovascular Disease

The etiology of cardiovascular disease (CVD) has been elucidated through the definition of risk factors in epidemiologic studies, notably the Framingham Study, initiated in 1948 (34). Interventional trials using relatively simple diet changes demonstrated rapid reduction of these risk factors and clinical benefits (35, 36). Interventional trials have demonstrated how a Mediterranean eating pattern significantly reduced cardiovascular events (37). More comprehensive interventions using a variety of lifestyle factors including diet have not only reduced but actually reversed existing coronary disease (38, 39), but these studies were small and of short-duration. In contrast to the notion that CVD occurs largely as a direct function of aging, research on the roles of dyslipidemia and inflammation creates new research opportunities for clinical and population-based interventions. Cardiovascular disease begins in childhood or even *in utero*, as demonstrated when mothers who have obesity give birth to children with thickened vasculature (40), perhaps even with early loss of lumbar arteries (41). Lifestyle medicine research should therefore prioritize children, as adult behaviors often begin in childhood.

There is emerging evidence to support the benefits of plant-based dietary patterns in primary prevention of CVD. Long-term epidemiologic studies have found that a healthful plant-based diet (that does not necessarily exclude all animal products) was associated with a significantly lower risk of type 2 diabetes and CVD (42). Small intervention studies have shown that a shift from a typical western diet to a vegan diet substantially lowered a atherogenic gut flora metabolite called trimethylamine-N-oxide (TMAO) which is induced by higher consumption of animal products especially red meat (43). Similarly, circulating lipoprotein (a) (Lp(a)), an established CVD risk factor that was previously thought to be genetically determined, has been shown to improve with plant-based diets (44). These observed health benefits of plant-based dietary patterns suggest other areas of inquiry that integrate basic, clinical, and translational research, especially when conducted within the cultural context of high-risk communities. Larger-scale, community-based interventional trials using adapted plant-based diets which are culturally sensitive to dietary traditions focusing on the African-American populations are underway (45).

#### Cancer

The World Cancer Research Fund and American Institute Cancer Research Fund have summarized evidence on cancer risk factors (46). The cancers most affected by diet and lifestyle behaviors are those of the gastrointestinal tract (e.g., colorectal) and hormonal cancers (e.g., breast and prostate). The Women's Health Initiative study (WHI) (47) was conducted to study cancer prevention and, while well-designed, utilized a weak dietary intervention. Thus, WHI yielded only a small difference in outcomes between the intervention and control arms and did not find significant benefits of the low-fat interventions on breast cancer incidence. However, a secondary analysis of WHI found that the low-fat pattern significantly improved overall survival among post-menopausal breast cancer patients (48). On the other hand, a clinical trial of breast cancer patients that dramatically increased consumption of fruits, vegetables, and fiber had no appreciable effect on breast cancer recurrence or mortality (49, 50).

#### Diabetes

Type 1 diabetes (“insulin-dependent diabetes” as the pancreas is unable to produce insulin) and Type 2 diabetes (“insulin-resistant diabetes” in which the body produces insulin but tissues are not able to respond to it) are both strongly linked to nutrition.

Excess adiposity is the strongest determinant of type 2 diabetes. The association of type 2 diabetes with dietary patterns is well-established, most notably the dramatic difference in type 2 diabetes prevalence in daily meat eaters when compared to vegans (51). At the tissue level, studies using magnetic resonance imaging have demonstrated lipid deposition in muscle and liver cells, leading to insulin resistance (52). Interventional trials using plant-based diets suggest that these diets can improve and potentially even reverse the course of diabetes (53).

The dietary etiology of type 1 diabetes needs to be explored further to test the various dietary factors that have been hypothesized to trigger the production of antibodies to insulin-producing cells including cow's milk (54), gluten, preservatives, such as nitrosamines, and low vitamin D levels. Clinical trials have not yet explored the role of plant-based diets for type 1 diabetes prevention and management. The association of prolonged breastfeeding with low rates of type 1 diabetes also needs to be explored further.

#### Autoimmune Diseases

Several studies have shown substantial benefits of plant-based diets for controlling rheumatoid arthritis, suggesting a role for dietary interventions not only for rheumatoid arthritis but also for auto-immune diseases more broadly (55).

#### Hormonal Conditions

Studies in the 1990's demonstrated that estrogen levels increase with high fat consumption and decrease with fiber intake, which is of particular relevance for breast cancer, dysmenorrhea, endometriosis, fibroids, and infertility (56).

#### Brain Health and Neurological Conditions

Observational studies (57) have demonstrated that a lower intake of saturated and trans fats is associated with greatly reduced risk of developing Alzheimer's disease. These findings are now being studied with the MIND Trial (58), among other studies using largely plant-based diets. Short-term dietary interventions have been shown to improve depression scores in both normal individuals (59) and those with depressive symptoms, the latter finding in both young (60) and general (61) populations. The dietary interventions typically reduce saturated fat and increase consumption of plant-based foods. Randomized trials have also supported a role for aerobic exercise in the treatment of depression in a dose-related manner (62). More clinical trials are needed both for cognitive and mood disorders.

#### Renal Disease

The beneficial role of plant-based diets in treating and potentially reversing chronic kidney disease is being explored. Kidney disease is particularly common among African-Americans and is mediated through diet and the *APOL-1* gene (63–65). The Adventist Health Studies have shown significant inverse associations between plant-based dietary patterns and risk of type 2 diabetes and total mortality among African-Americans (66), suggesting the possibility of benefit for reducing renal failure in this population.

#### Summary

In summary, strong, well-designed and -powered intervention studies using appropriate comparator groups are needed to advance the understanding of plant-predominant eating on the prevention and treatment of a wide array of diseases. High priority populations include children (41) and understudied, underserved, and disadvantaged populations with highest incidence of multiple diet-impacted disease. Emphasis should be placed on human rather than animal studies.

Studies should also focus on our current understanding of diet quality (52, 67) considering the quality of fats, carbohydrates, and protein not merely their relative proportions. Sources of animal- vs. plant-based proteins and high quality low or high fat, low or high carbohydrate consumption, and quality of overall diet need to be specified more precisely in study designs. Large cohort studies have shown that long-term consumption of plant-based (as compared to animal-based) protein is associated with lower all-cause and cardiovascular disease mortality (68, 69). The associations of low-carbohydrate diets (LCDs) and low-fat diets (LFDs) with mortality may depend on the quality and food sources of macronutrients rather than the carbohydrate or fat proportionate content alone (70).

Plant-predominant nutrition research priorities appear in Table 1.

**Table 1:** Nutrition research priorities.

**Disease**	**Methodology considerations**
Cardiovascular Disease	Translational studies particularly in children Counter disinformation campaigns and studies
Cancer Prevention and Survival	Prevention and survival studies (e.g., diet + exercise)
Diabetes: Type 1 and 2	Type 1: Prevention: dairy intake and relationship to type 1 DM onset Type 2: Intervention trials with plant-based diets Translation, complication prevention and reversal: ocular, neurological, and renal
Autoimmune diseases • Rheumatoid Arthritis • Type 1 diabetes • Psoriasis • Multiple sclerosis • Thyroid conditions • Asthma • Sjogren's disease	Large clinical trials powered with strong interventions to build on abundant case histories of remission or reversal
Hormonal Diseases Thyroid disease: iodine, autoimmune disease Hashimototo's and Graves disease antibodies	Large clinical trials to build on case studies
Brain health and neurological diseases • Mood disorders • Depression • Attention-deficit disorder	Large clinical trials to build on case studies and smaller studies
Renal	Clinical trials for stopping progression and reversing chronic renal failure
Diet quality and cultural aspects of plant-forward nutrition	More comparisons of clinical effects and outcomes of animal vs. plant-derived diets and analysis of micronutrient composition

### Physical Activity Overview and Prioritized Research

Physical activity is recognized as a key behavior related to the prevention and treatment of many chronic diseases and other health-related conditions. Exercise is associated with mitigation of stress and depression (71). The evidence to support the health benefits of physical activity have been accumulating for decades. Some of the early research to support the health benefits of physical activity came from observational studies, such as studies of transit employees or other types of occupations (72). These studies showed that individuals with more occupational physical activity had lower risk of mortality and some morbidities than those individuals in more sedentary occupations. These observations provide a foundation of scientific study to support the importance of physical activity as a key lifestyle behavior to promote health and well-being.

There have been key publications and reports over the past few decades that summarize the evolving list of health benefits of physical activity along with key contemporary considerations for the promotion of a physically active lifestyle (73–75). For example, the initial Surgeon General's Report and the 2008 Physical Activity Guidelines stated that physical activity should be accumulated in bouts of at least 10 min to achieve an average of 30 min per day of moderate-to-vigorous physical activity (73–75). In addition to common recommendations for the amount of physical activity that may be needed to improve health, guidance on how to achieve this amount has evolved over time. The 2018 Physical Activity Guidelines Advisory Committee suggested that all moderate-to-vigorous physical activity, regardless of the length of bout in which it was accumulated, contributed to the potential health benefits (75).

Another important area of study involves sedentary behavior, mostly in the form of sitting, and how it contributes to poor health. Research now suggests that sedentary behavior may have negative influences on health that are independent of participation in moderate-to-vigorous physical activity. This has resulted in new recommendations that persons should sit less and move more to improve health (75).

#### Molecular, Cellular, and Aging Mechanisms

Understanding physical activity-induced effects on molecular, sub-cellular, cellular, tissue, organ systems, and intra-systems (e.g., cardiovascular and central nervous system) can now be pursued (76) (Figure 1). The cellular and molecular mechanisms of physical activity-induced health benefits are becoming better understood (77). Comprehensive models are being created that include both inherent (genetic and epigenetic) and acquired factors (age, disease state, environment, fitness, and nutrition), which together determine individual differences in how physical activity impacts health and disease (Figure 1).

Why we age and the rate at which we age, i.e., chronological age vs. biological age or lifespan vs. healthspan—are determined by multiple molecular processes. Exercise stimulates stem cell self-renewal in brain, muscle, and other tissues (78). Telomere shortening, bioenergetics, mitochondrial function, and a variety of pleiotropic effects affect biological aging and the development of age-related conditions. The exploration of “exerkines” and epigenetic mechanisms may yield insights into the signaling pathways that connect exercise and cognitive function as well as other observed improvements (79, 80) (Figure 1).

Better understanding of the factors that promote adopting and sustaining health behaviors generally, and regular physical activity specifically, is needed. Exercise and physical activity are more likely to be incorporated into daily living when tied to one's personal life goals, mission, aspiration, and purpose. *Health coaching* (81) and the use of digital or other trackers, feedback and social support either in person or through virtual groups have been shown to improve initiation and reinforcement of activity and healthier behaviors. Long-term healthy habit improvement and adoption are most likely when incremental small steps are introduced, achieved, and reinforced (82). Other important factors to increase successful engagement include clinician modeling of desired behaviors and a clinical systems approach using multi-disciplinary teams, the “prescription” of lifestyle behaviors, and support which elevates the importance of their therapeutic use and impact as embodied in “exercise as medicine” (81, 83).

#### Summary

Physical activity research priorities appear in Table 2.

**Table 2:** Physical activity research priorities.

**Question/Priority Area**	**Methodology Considerations**
Physical activity and sedentary behavior on multiple health outcomes across the lifespan	Dose response, role and contribution of light-intensity vs. moderate-to vigorous physical activity Independent and interactive effects Is effect modified by demographic factors, including sex and race/ethnicity?
Biological/molecular/cellular responses to physical activity and sedentary behavior	Anti-inflammatory effects of mechanical stretch; Stimulation and physical stimuli to enhance the regenerative cascade Molecular tenets of exercise or physical activity, e.g., mechanotransduction Translation of mechanical to molecular cellular response Neurochemical measurement Measurement of mitochondrial function Measurement of epigenetic effects Computational analysis and *in silico* modeling
Effective intervention strategies for increasing physical activity through actions in multiple settings, across the lifespan	Larger trials to determine how the effectiveness of interventions differs by sex, age, race, ethnicity, socioeconomic status, and other factors Machine learning analysis of trial results to define novel biomarkers or mechanisms Individual cohort identification and application of precision medicine approaches

### Sleep Overview and Prioritized Research

The sleep/wake cycle is the most enduring and obvious manifestation of the circadian timing system which can influence inflammation, immune dysfunction, oxidative stress, and epigenetic modification, all associated or causally related with the onset and disease (Figure 1). Sleep is defined as a recurring, reversible, neuro-behavioral state of relative perceptual disengagement from and unresponsiveness to the environment. Sleep optimizes adaptation and function at every level of biological organization: molecular energy function, cellular, tissue, circuit organ, organ system, systemic physiology cognition and affect. Sleep and wakefulness exist within the 24-h rhythms that organize almost every aspect of our physiology. Every cell has a circadian clock that rests on a transcription-translation feedback pathway involving nine core clock genes which regulate their own transcription every 24 h (84). The “master pacemaker,” the suprachiasmatic nucleus of the hypothalamus located above the optic chiasm, receives light-dark inputs (85). Endogenous rhythmicity occurs within cells determining such functions as core body temperature and cortisol production which vary over 24-h periods. Performance, which varies during the course of each day and among individuals, can be expressed as “chronotype” *viz* the preference for evening or morning hours. Sleep can be measured by self-report, actigraphy, electrophysiologic sleep studies (86), brain imaging, and even in cultured tissue outside the body (87). Circadian variation of gene expression is not only important for disease but also for therapeutics of those diseases (88). The circadian timing system impacts, is impacted by, and receives feedback regulation from health behaviors, such as physical activity, sleep, eating, and light-dark cycles (89). These clocks in the brain and cells regulate brain and body function that determines physiology, behavior, and performance.

Behaviors change the timing of the clock, and in turn the clock affects all of the health behaviors that lead to disease. Sleep ultimately optimizes function at every level of biological organization from molecules to entire organisms. Sleep provides protection from oxidative stress (90) and clears the brain of waste and breakdown materials that accumulate during wakefulness. The so-called “glymphatic system,” a lymphatic system for the brain, helps clear beta-amyloid and other proteins in the cerebrospinal fluid interstitial spaces (91, 92). Aging reduces glymphatic clearance, which is associated with the accumulation of beta amyloid protein and other proteins associated with Alzheimer's disease, which is also marked by severe sleep-wake and circadian dysregulation (71, 93). Food consumption patterns and timing entrain circadian rhythms and metabolism and can be tied to weight gain and obesity (94, 95). Sleep restriction increases appetite and reduces insulin sensitivity (96). Time-restricted feeding may help prevent obesity and promote weight loss (77, 97–99).

Sleep health is characterized by regularity, efficiency, duration, timing, alertness, and satisfaction. Regular sleep of sufficient duration, efficiency, timing, and quality is related to health and disease. Sleep disorders, such as insomnia, sleep-related movement conditions, parasomnias, and central nervous system-mediated and breathing-related abnormalities, such as obstructive sleep apnea have been shown to be associated with health risks. Obstructive sleep apnea has been most notably associated with cardiovascular risk (hypertension, coronary artery disease, stroke, and atrial fibrillation), cardiometabolic risk (obesity and diabetes) (100), and neuropsychiatric risk (sleepiness, inattention, impaired cognition, and depression). Furthermore, a meta-analysis suggests a positive association between insomnia and future depression (101), and other studies suggest that sleep duration is inversely correlated with adolescent suicidal behaviors (102).

Circadian rhythms and sleep patterns (amount, regularity, timing) should be proactively leveraged and promoted to improve health and mitigate diseases which can be exacerbated by sleep disturbances and the metabolic changes they produce. Use of a broader socio-ecological model which incorporates environmental changes, policies and practices to improve recognition of circadian rhythm, essential nature of sleep, and health (103–105) is important and likely to be clinically impactful.

#### Summary

Sleep research priorities appear in Table 3.

**Table 3:** Sleep research priorities.

**Question/Priority area**	**Methodology considerations**
**BASIC SCIENCE**	
• Circadian and sleep-related control of gene expression and regulation • Sleep-related regulation of CNS interstitial protein clearance • Restorative function of sleep at the molecular, cellular, and physiological levels	• Application of cutting-edge neuroscience techniques (e.g., optogenetics, CRISPR) to circadian and sleep questions • Refined model systems, including *in silico, ex vivo*, and *in vivo* animal models
**TRANSLATIONAL SCIENCE**	
• Effect of circadian rhythms and sleep on common therapeutics (e.g., chronopharmacology) • Intersection between sleep/circadian physiology and metabolism at the molecular, cellular, and physiological levels	• Simple, accurate, reliable methods to identify circadian phase and amplitude in intact organisms, including humans • Development of high-throughput assays and methods for examining drug effects in a circadian context • Methods for measuring sleep and circadian-related aspects of CNS interstitial protein clearance and metabolism
**CLINICAL AND IMPLEMENTATION SCIENCE**	
• Novel measures to identify the relationship between sleep disorders (sleep apnea, insomnia, restless legs syndrome), sleep treatments, and health outcomes • Dissemination of behavioral sleep interventions • Novel sleep and circadian interventions to improve health and function at the individual, community, and societal levels • Clinical practice guidelines for induction and sustainment of healthy sleep behaviors	• Improved measures of sleep apnea that relate more closely to health and mortality outcomes • Improved methods for simultaneous measurement of sleep and brain function (e.g., functional connectivity and region-of-interest activation) • Refined measures of sleep and circadian rhythms using remote monitoring and passive sensing • Novel delivery methods and systems for behavioral interventions • Incorporation of sleep and circadian rhythms into multiple health behavior change programs • Improved communication between sleep/circadian and lifestyle medicine communities

### Stress Overview and Prioritized Research

The acute stress response is adaptive and helps activate the cascade of biological and physiological responses to actual or perceived threats. This response is triggered autonomically to help remove people from danger and enhance survival. With prolonged or extreme stress, brain and body responses can become overwhelmed, and physiological changes due to chronic stress increase the risk of physical and mental conditions, such as cardiovascular disease, irritable bowel disease, obesity, and depression (106–108) (Figure 1).

Depression is a multi-determined disease with rates of the disorder and related disability and costs on the rise worldwide. Biological (e.g., genetic, biochemical, hormonal, inflammatory) and environmental (e.g., psychosocial adversity) have been implicated as predisposing, precipitating, and perpetuating factors (109). Depressive disorders have shown increased prevalence and in turn increased the incidence of many chronic physical diseases including asthma, arthritis, autoimmune diseases, cardiovascular disease, cancer, diabetes, neurological disorders, and obesity (110–112). The biological underpinnings, such as autonomic dysregulation and inflammation shared across depression and many of these conditions, serve as targets for stress reduction strategies with antidepressant effects.

One's ability to reset the stress response to a homeostatic resting state is called *allostasis* and the toll of maintaining this adaptive capacity over a lifetime is the *allostatic load* (113). *Resilience* is the degree to which people can cultivate adaptive responses by reducing negative effects of stress and returning to a state of healthy well-being (114, 115). One of the most powerful mechanisms to reduce stress and enhance resilience is by eliciting a relaxation response (116). Mind-body therapies (MBT, such as mindfulness meditation, breathing exercises, yoga, etc.) and cognitive behavioral therapy (CBT) can elicit relaxation and improve negative brain-body effects of chronic stress while maintaining reactivity to acute stress (117). Mindfulness training fosters attention and acceptance toward one's present moment experience. CBT teaches adaptive coping by reappraisal of (negative) thinking and changing maladaptive behaviors. Cognitive appraisal can be a powerful mediator of perceived stress (71). With regular practice, these types of MBTs enhance both reactive and anticipatory resilience and have been associated with improved immune function and reductions in chronic pain, cardiovascular disease, anxiety and depression (118–121). The growing availability of these behavioral interventions by digital technology has greatly increased their accessibility and reach worldwide (122).

Notably, it is important to create better understanding, definition and dissemination of a framework for understanding, research, and practice that distinguishes “stress” from the “stress response” (113, 123–125). In this regard, there are emerging molecular studies in the MBT field that tie into the lifestyle medicine research priorities described above (Figure 1). For example, recent epigenetic research (126) on the positive role of meditation in rapid, epigenetically driven protein expression to reduce IL-6 (127) suggest the need to study the link between MBT's and inflammation (Figure 1).

The current COVID-19 pandemic has occurred in the context of stress associated with issues of political, socioeconomic, and racial divisions that is unusual in both scope and intensity (125). Perhaps at no time in recent memory has the ability of individuals, families, and communities to recognize and proactively address mental, physical, and emotional adverse effects from these “acute” catastrophes (COVID-19 illness, mortality, social and economic effects) and “chronic” life-shortening institutional biases (racism, poverty, social disparities) been more needed.

#### Summary

Stress research priorities appear at Table 4.

**Table 4:** Stress research priorities.

**Questions/Priority area**	**Methodology considerations**
Heterogeneity in how stress/allostatic load paradigms and indices of adaptation/resilience are defined.	Consensus determined consistent definitions and measurements across multiple domains. Movement from efficacy to effectiveness. research from small samples to populations.
Lack of standardization of MBTs across studies and mediators of their positive effects (e.g., physiologic, biochemical, brain and body organ level, immune, microbiome, epigenetic, psychological, behavioral).	Determination and standardization of “active” components of MBTs. Consistent domains of biological and psychological outcomes and mediators. Understanding of processes and interrelationships at multiple levels: genes, cells, organs, networks, and organism.
Differentiation of short-term and long-term outcomes in stress reduction and increased resilience and differentiating response from remission and relapse prevention.	More longitudinal studies. Machine learning analysis of trial results to define novel biomarkers or mechanisms.
Better incorporation of health-technologies to increase access to MBTs, scalability, and predictive capacity to identify at-risk populations to move from treatment to prevention.	Evaluation of effectiveness of technology-enhanced behavioral interventions. Machine-learning for better predictive analytics to personalize interventions.

### Substance Abuse and Addiction Overview and Prioritized Research

Tobacco use remains the leading cause of U.S. preventable deaths. Despite decades of successful public policy and clinical efforts, thirty-four (34) million Americans still smoke and two-thirds report a desire to quit. Our collective challenge remains to deploy proven effective policy, environmental health and clinical solutions to reduce smoking and undertake targeted emerging basic science and behavioral research to better understand nicotine addiction and accelerate our progress to date (128).

Recent social, economic, and environmental factors have fueled the national rise in substance abuse generally and the opioid epidemic, specifically. Misuse of alcohol and other substances are common in the U.S. with ~7% of the population meeting criteria for a substance use disorder (SUD), 19% using illicit substances, 6% misusing prescription medications, and 6% consuming unhealthy amounts of alcohol (126). Despite the prevalence, those with a SUD have traditionally been excluded from the healthcare system and relegated to a disconnected collection of acute care specialty treatment providers, with limited access to either medical care or medications for SUD. The opioid epidemic has revealed the limitations of the disconnected SUD treatment system, as many people with an opioid use disorder (OUD) have died while cycling between hospitals, jails, or emergency medical service (EMS) contacts and without access to evidence-based treatment, including lifesaving medications (129).

Integrating people with an OUD as well as other SUDs into the healthcare system is the solution to reducing mortality and morbidity associated with this population. Individuals with an OUD are like other patients with chronic health disorders; they need ongoing care within a primary care patient-centered medical home (PCMH) as well as easy access to the array of specialty treatments and services to address the sequelae of infections, organ damage, and socioeconomic loss that result from their chronic illness. The rapid application of agonist medications, including methadone and buprenorphine, could reduce the OUD mortality rate by 50%, which translates to over 25,000 lives saved each year (129).

A four-stage cascade model of care has been recommended as a framework for engaging and retaining people with an OUD, based on the protocol used to identify and rapidly treat individuals infected with HIV (130). Individuals can be identified in hospitals, emergency departments, jails, EMS, needle exchange centers or homeless shelters (stage 1), where rapid access to buprenorphine can lead to 70% or more patients engaging in treatment, upon discharge (131–133). Effective treatment (stage 2) includes rapid access to medication-based treatment and other psychosocial services (134). Providing a community-based recovery coach or case manager can improve the transfer rate from crisis to medication-based treatment (135, 136). Stepped care procedures, such as the hub and spoke or collaborative care models (stage 3), are used to modify a person's OUD medications, medical care and psychosocial needs over 6 or more months, using the same approach that primary care practices (PCPs) apply to chronic diseases. Innovations in primary care, such as value-based payments for improved outcomes, expansion of telemedicine technology (137), embedding recovery coaches in PCPs (138), and measurement-based care (139) have all been found to improve long-term outcomes for people with an OUD (stage 4).

Finally, it is important to leverage existing innovative and integrative research funding programs, such as the NIH OppNet, which seeks to foster collaboration across NIH to accelerate discoveries in basic behavioral and social sciences research (including addictions) (140).

#### Summary

Substance abuse and addiction research priorities appear in Table 5.

**Table 5:** Addiction research priorities.

**Question/Priority area**	**Methodology considerations**
**PREVENTION OF SUD**	
What are the physiological markers, genetic history, adverse childhood experiences, socioeconomic factors or co-occurring conditions (chronic pain or depression) that increase an adolescent's risk to a SUD and how could these measurable factors be weaved into proactive skills training	Prospective research that can both identify risk of SUD as well as intervene before onset for those with known risk factors, such as adverse childhood experiences or emerging risks, such as exposure to prescription medications in the home
What are the potential skills training and lifestyle medicine interventions that young people can enhance though grade school or college, if their risk of an SUD can be identified prior to exposure to alcohol and other substances	Expand research, using longitudinal timeframes, on cognitive skills training, such as improving executive functioning or strengthening emotional regulation in adolescents and young adults to assist them in effectively navigating through the exposure of alcohol and other substances
**EARLY DETECTION OF MISUSE PATTERNS OR EARLY ONSET OF SU**	
How is substance misuse interrelated with other lifestyle factors, such as the quality of sleep, exposure to stress, social support, nutrition, physical activity or sense of safety	Develop interventions that can measure and enhance a person's social and physical environments for those who already have high risk markers, such as adolescents or college students exposed to stress behaviors
How can technology be used, such as fit-bit like devices or smart-phone apps to provide individuals with real-time messaging, nudges or warnings around urges to use alcohol or other substances or identify relapse risks in time for effective problem-solving strategies	Expand the use of self-guided technology, phone-based applications, and web-based interventions for individuals with misuse patterns, who would likely benefit from low-touch interventions and do not need high-touch services, such as a SUD outpatient
Can big data be harnessed for real-time predictive modeling of patients who are at risk of or already displaying unhealthy behaviors around substance use or showing signs of prescription medication misuse	Explore the potential of machine learning for rapid identification of SUD risks that can be used for proactive interventions; so far, the research has been used on historical data sets, but has yet to be applied to early interventions
Can lifestyle medicine be applied to the treatment of people with chronic pain, such as expanding interventions around nutrition, physical activity, stress, sleep, social connection both before and after the onset of an OUD	Develop and test value-based payments and other alternative payments to expand the use of lifestyle medicine interventions in the treatment of chronic pain, as there appears to be a need for a cultural and financial change in how people with chronic pain are treated over time
Is substance misuse a problem or a solution to solving another problem, such as managing physical or emotional pain. stigma, social disconnection or caused by impulsive and adversarial behaviors (frontal lobe dysfunction or immaturity plus chemical effects)	Identify personality traits, such as impulsiveness and sociological factors, such as stigma or social isolation, that can be used in developing alternative treatments for youth who are at risk of a SUD
**TIMELY APPLICATION OF EFFECTIVE TREATMENTS FOR INDIVIDUALS WITH A SUD—EXAMPLE OUD**	
How can the impact of stigma be reduced across the healthcare system, including its impact on racial minorities, pregnant women and those being discharged from incarceration who have an OUD	A research priority is measuring institutionalized stigma associated with people who have an OUD or other SUD and how stigma is hindering the application of effective treatments or increasing disengagement from effective care
Can we develop flexible, stepped SUD treatment interventions that can be modified over several months, based on a patient's response to each incremental stage of treatment	Develop RCTs that include a two-staged process to accurately assess the incremental impact of psychosocial interventions beyond medications, based on an individual's response to medications and TAU impact,
How can we design flexible, integrated models of care for people with an OUD using effective protocols in healthcare, such collaborative or measurement-based care, or telehealth and e-therapy that can both engage individuals over years while providing care in either office or home-based settings	Identify metrics, technological monitors and telehealth interventions that can be used for effective triage of SUD treatment; these metrics or technologies could be used for both triage as well as for home-based monitoring or treatment for those living in rural locations
What is needed in treatment funding or innovation to weave in the healing powers of the community, including the digital support network that individuals with a SUD could utilize to improve outcomes.	Deign research projects as well as alternative funding models that can measure and incorporate elements of recovery capital, including the growing network of digital social support (DSS) to improve SUD outcomes

### Positive Psychology and Social Connectivity Overview and Prioritized Research

Positive psychology interventions have been shown to improve subjective well-being, which is associated with improved health behaviors, health outcomes, and longevity. The upward spiral theory of lifestyle change suggests positive emotion promotes healthy lifestyle choices (141). A meta-analysis of a spectrum of positive psychology interventions, such as the practice of gratitude, forgiveness, and savoring shows a small, but statistically significant, impact on psychological and subjective well-being, which can, in turn, be associated with physical health benefits (142). Other studies have shown an association between having a sense of life purpose and meaning and higher use of preventive services, as well as lower CVD mortality in those who already have the disease (143) (Figure 1).

Of the positive psychology pillars (positive emotion, engagement, relationships, meaning, and accomplishment), social connectivity has the most powerful health benefits in long term cohort studies. These include the ongoing, eight-decade cohort in the Harvard Study on Adult Development, which has conducted analyses controlling for many confounding variables (144, 145). Groups with close social ties tend to live longer than those with similar risk conditions but lacking social ties. Conversely, social isolation (such as living alone, having a small social network, participating in few social activities, feeling lack of social support and loneliness) is associated with greater all-cause mortality, increased morbidity, lower immune system function (likely linked to chronic inflammation), depression, and cognitive decline (146–148) (Figure 1). Interestingly, recent research on micro-moments of connectivity during human interactions with strangers, as well as friends, demonstrates a boost in the parasympathetic nervous system, the “tend and befriend” response, with correlated physiologic benefits (149, 150).

What is the “active ingredient” of effective positive psychology interventions? Further study is needed to uncover the biological and physiological mechanisms of action and nuanced effects of these types of interventions, including social connectivity, in populations of different demographics and cultures at both the individual and community level. Also, the impact of in-person vs. digital technology delivery channels, optimal “dosing,” “person-activity fit,” and the nuanced effects of high arousal vs. low arousal emotional states require careful study. These studies of proximal outcomes should be incorporated and tested in non-mental health care settings (e.g., lifestyle medicine and primary care), as well as mental health care settings, to develop a robust evidence-base of effective interventions for medical practitioners and health teams. The field of lifestyle medicine that aims to treat and reverse, as well as prevent, lifestyle-related diseases through comprehensive lifestyle interventions is in a prime position to help build the evidence-base for applying positive psychology and social connectivity interventions in health care.

#### Summary

Positive psychology and social connection research priorities appear in Table 6.

**Table 6:** Positive psychology and social connectivity research priorities.

**Questions/Priority area**	**Methodology considerations**
Basic Science: Biological and physiological mechanisms of actions underlying specific positive psychology interventions (PPIs) and social connectivity	Basic science, Big Data, and *in silico* modeling Considerations: Consensus determined consistent definitions and measurements across multiple domains Active ingredients of positive psychology interventions and social connectivity
Clinical Science: Physiological, neurological, immunological, psychological, epigenetic and microbiome outcomes of key positive psychology interventions (e.g., positive emotions, engagement, relationships, meaning and accomplishment) when used as part of well-being maintenance and lifestyle medicine treatment in health care settings	Clinical trials, longitudinal and cohort studies, big data and modeling Considerations: Consensus and standardization of terminology for emotional states and psychological outcome measures Consistent distinction of cognitive self-appraisals and emotional states Cultural, age, and race differences in the health effects of high arousal vs. low arousal positive affect, and cultural nuances in benefits arising from different PPIs, e.g., gratitude practice and acts of kindness Health effects of teachable positive psychology-based skills, such as cultivating hope and optimism vs. natural, personality or genetic based predispositions in affect Effective strategies for identifying the best person-activity fit and optimal “dose” of positive psychology interventions Differences in health effects social isolation vs. loneliness and impact of expectations The short- and long-term health and well-being effects of social media engagement
Clinical Science: Emotional and mental health effects of each pillar of a healthy lifestyle, as part of lifestyle medicine	Types of studies: Clinical trials, longitudinal and cohort studies, big data and modeling Considerations: Bidirectional and reinforcing effect and mechanisms of action between healthy behaviors and positive emotions Effective social interventions for increasing health behaviors and improved physiologic outcomes
Population-Level Science: Nature of community and technological environments that promote and support the beneficial health effects of positive psychology-based activities and social connectivity	Types of studies: Considerations: Cohort studies, Big Data, modeling and AI Evaluation of effectiveness of public health interventions, environmental redesign, and technology-enhanced behavioral interventions Machine learning for better predictive analytics to personalize interventions.

## Socioecological Influences, Environment, and Exposures

There is a need to move beyond framing individual lifestyle behaviors as only personal “choices” toward using an integrated model of socioecological influences on health, within which clinical application of lifestyle medicine is a foundational best practice component. The term “lifestyle medicine” could, if taken in isolation, imply that an individual clinical approach could singularly or adequately treat and reverse chronic diseases which are strongly influenced by a variety of socioecological factors (151). Many are beyond the control of individuals and are subject to public policies, economic trends, social inequities, and other macrostructural influences.

Every patient and family exists within, and is supported (or constrained) by, specific and different environments that are increasingly defined by culture, location, employment, education, housing, air, water and community. Not surprisingly, then, human health, well-being, performance, and environmental health are closely connected. Built and natural environments (sidewalks, parks) are needed to support safe physical activities which become part of regular, easy-to-do routines at home, in schools, at worksites and in neighborhoods (152). There is growing evidence that social and environmental inequities experienced by some racial and ethnic groups and underserved populations contribute to increased rates of a variety of diseases (153). Inequities in the social determinants of health (many operating across generations), contribute to health behaviors and outcomes across population groups and geographies. We suggest that the imperative to address not only the social but also the ethical determinants of health should underlie all initiatives to improve health, increase access to medical care, and create better outcomes (154).

Environmental conditions, both indoor and outdoor, have been linked to objective measures of the stress and relaxation response, such as heart rate variability and cortisol levels. In striking recent studies, environmental conditions have been associated with epigenetic changes in children living in disadvantaged neighborhoods (155, 156). As most Americans currently spend over 90% of their time indoors, the critical role of building design and environmental conditions on cognitive function, sleep, stress and productivity is increasingly being realized (157, 158). This role has become especially apparent during the recent period of enforced, long-term lockdowns due to the COVID-19 pandemic. Improved indoor air quality utilizing frequent ventilation changes which lower CO_2_ and volatile organic compound (VOC) levels are among the measures outlined in the Living Building Challenge certification (159). Additionally, sustainable sourcing of building materials, natural light access, and energy efficient heating/cooling systems are required. These measures often invoke the concept of “Biophilia” (160)—humans' innate connection to nature- and biophilic design (161). This, a deliberate design paradigm intended to foster an affinity in the built environment produces measurable improvements in health, healing and pain management (162, 163). It may ultimately produce healthier, happier, and more productive individuals and populations in the home, school, workplace, and neighborhood. Green building certification, such as the WELL building certification (164) measures even more directly how the indoor environment affects human health and provides guidelines for improving occupant health outcomes.

Growing scientific and medical evidence of the health impact of environmental chemicals builds on the troubling legacy of dichlorodiphenyltrichloroethane (DDT) and diethylstilbestrol (DES). Research over the past two decades has consistently identified that minute levels of a broad suite of synthetic chemicals can disrupt endocrine pathways and thereby contribute to disease and disability, including at low levels of exposure in susceptible windows of vulnerability.

The accumulating evidence is strongest for four categories of chemicals: flame retardants used in furniture and electronics; pesticides used in agriculture; phthalates used in food packaging, cosmetics, and personal care products; and bisphenols used in aluminum can linings and thermal paper receipts (165). In addition, at least 1,000 synthetic chemicals can disrupt endocrine (including insulin and thyroid hormones) and reproductive functions (testosterone, sperm count, polycystic ovary syndrome, endometriosis and fibroids), increase the risk of obesity and impact human development (e.g., IQ). Better understanding of risk and exposure relationships is fundamental to advancing prevention and intervention strategies. Of particular concern are agricultural workers exposed to the highest levels of pesticides and children most impacted by exposure to environmental lead and pesticides in local drinking water. Population-based policy solutions, akin to removing lead from gasoline must be applied more often and broadly. The EAT-Lancet Commission report “Eat, Food, Planet” related greater adoption of plant-based dietary patterns and improved agricultural practices to enhanced environmental sustainability (166). Finally, while beyond the scope of the Summit, we note that lifestyle practices are clearly affected by the most glaring, overarching environmental concern leading to multiple adverse health effects, namely climate change.

## Underserved and Understudied Populations and Perspectives

Women, children, and marginalized populations have been excluded from much research. Most common chronic diseases occur later in life and thus research has largely focused on older adults. However, the foundations of lifestyle behaviors are set early in life providing an opportunity to create an integrated approach to the full continuum of women's and children's health. A lifecourse approach can encompass pre-conception, pregnancy, infancy, childhood, and adolescence in ways that will influence health later in life. Preparation for pregnancy and pregnancy itself (167) represent the ideal “teachable moment” for assisting women and families to understand the foundation of healthy living and to develop and adopt life skills to promote optimal growth and development which can span multiple generations. Pre-conception health “primes” not only maternal health (e.g., avoiding excessive weight gain and pregnancy complications) but also affects embryonic development and growth that are key drivers for the development of congenital malformations and intrauterine growth restriction. During pregnancy and the perinatal period, inadequate plant-based nutrition, lack of folic acid supplementation, stress, environmental toxins, smoking, alcohol, and endocrine disrupters can adversely affect pregnancy outcomes and subsequent children's disorders. Alterations to the epigenome and germ cells mediate these health and transgenerational effects (168) (Figure 1). Maternal exposure to environmental chemicals and their transmission in breast milk to the infant are beginning to be studied (169). Plant-based nutrition, providing a diet rich in micronutrients, as well as regular physical activity are now seen as essential for producing healthy pregnancies and babies.

“Toxic stress” in early childhood from psychological trauma, physical abuse or adverse child experiences is now known to affect neurological development at the molecular level and increase the risk for future medical and psychological chronic disease (170, 171). Poor childhood lifestyle behaviors often become manifest in preventable conditions involving multiple organ systems. These behaviors drive overuse of the medical system and numerous specialty consultations when root causes are either overlooked or unaddressed. Lifestyle change is more likely to occur and be impactful during younger childhood when family dynamics can be leveraged, as opposed to during adolescence, which is often characterized by rebellion. Methodological challenges to conducting pediatric obesity studies have been described (172). Additionally, treatment of children with serious medical conditions must concurrently address psychosocial and lifestyle-related needs and behaviors of the patient and their family as their traumatic experiences can negatively affect medical compliance and long-term outcomes.

Racial, ethnic, and cultural groups have specific strengths, challenges, and beliefs that should be recognized, leveraged, and addressed. Foundational to a whole person- and family-centric approach is acknowledgment of the chronic stress and physiological effects which accompany the sense of discrimination and bias.

We need to better understand and leverage the sociocultural aspects of purpose, spirituality and religion which are powerful forces aligned with healthy behaviors and have not been well-studied. Many religions, for example, in addition to providing stress relief, emotional and spiritual comfort, have emphasized healthy living through eating plants rather than animals. However, the study of how to leverage these specific values and messages in the context of religious and spiritual belief systems and cultures is lacking.

Optimizing the economic value of health and productivity (173, 174)—particularly among large, self-insured employers—represents an important route to demonstrate the “business case” for lifestyle medicine. Extending beyond clinical and health outcomes to include safety, absenteeism and productivity, the Total Worker Health framework created and promoted by the National Institute of Occupational Safety and Health (NIOSH) (175) represents research opportunities to assess the impact of chronic disease treatment and reversal through clinical practice, particularly through the use of on-site clinics and lifestyle medicine (176). The occupational productivity impact of sleep quality and insomnia was recently reviewed and summarized (177). A recent national health and economic analysis of limiting BPA in foods demonstrated a significant reduction in childhood obesity, heart disease, and cost savings (174). Similarly, recent large, US-based population analyses which model the national economic impact of adoption of healthy, plant-based eating can be shared and extended to other lifestyle domains as the basic and clinical science continues to emerge (178).

## Applying Appropriate Strength of Evidence Methodologies to Lifestyle Medicine Interventions

The Summit reviewed strength of evidence methodologies and discussed whether a new framework would be more appropriate to measure the impact of applying clinical lifestyle treatment and reversal interventions to reduce disease and improve healthy aging (Figure 1). Current methods for assessing strength of evidence (SOE) prioritize the contributions of randomized, placebo-controlled trials (RCTs). However, RCT's may not always be suitable to study lifestyle interventions for improved longevity, vitality, or successful aging. Assessment of evidence relevant to lifestyle medicine requires adaptation of SOE approaches when outcomes and/or exposures obviate exclusive or preferential reliance on RCT designs. SOE tools, such as the Hierarchies of Evidence Applied to Lifestyle Medicine (21) are needed to accelerate and prioritize impactful lifestyle-based clinical research and practice deployment.

## Moving Forward: Focusing and Accelerating Public and Private Sector Research to Treat Root Causes of Chronic Disease

In the coming years, federal biomedical funding priorities via the NIH are likely to be targeted to precision medicine, genome editing, the brain, and cancer immunotherapy (with foundational emphasis on immunology and inflammation). The recently announced 2020–2030 Strategic Plan for NIH Nutrition Research (179) highlights the systems approach to addressing molecular, behavioral, and societal factors in the prevention and management of diet-related diseases.

Non-federal research-supporting organizations, such as the American Heart Association (AHA) will also have a significant role in supporting new knowledge generation around lifestyle and chronic disease prevention, treatment, and reversal. The AHA's “Life's Simple 7”: not smoking, healthy eating, physical activity, healthy weight, blood pressure, cholesterol, and blood glucose are already emphasized (180). Adding healthy sleep would create a “Life's Essential 8.” The AHA's promotion of Big Data and Precision Medicine as well as accelerating progress and breakthroughs through using interdisciplinary consortia (e.g., the Strategic Focus Research Networks) to focus on specific conditions, issues and populations can be re-visited given progress in basic, clinical and population sciences.

The U.S. military has focused on eight interrelated components in the DoD Total Force Fitness framework (181), which mirrors lifestyle medicine domains and informs the Department's research priorities. The U.S. Special Operations Command Preservation of the Force and Family program, one of the first lifestyle medicine-focused initiatives in DoD emphasizes service and family member health, well-being and performance (182). Importantly, the military understands that care providers must be embedded in military units which may include physical therapists, occupational therapists, and potentially also human performance coaches. The U.S. Veterans Health Administration, also prioritizes the broad spectrum of well-being by including a focus on spirit and soul in caring for veterans, as encompassed in the Whole Health aspirational framework (183). This approach has had a favorable impact not only on patients but also on doctors, who report a much lower sense of burnout.

The COVID-19 pandemic has exhibited increased severity and mortality among those who are socioeconomically disadvantaged, including African-American, Hispanic/Latino, Native Americans, and those with multiple co-morbidities (184, 185). The increased risk for both transmission and severity once infected is multifactorial including the inability to shelter at home, need to work in service jobs with heightened exposure to the virus, and living with socioeconomic disadvantages often caused by a history of systemic marginalization and exclusion. Living or working in overcrowded conditions without access to COVID testing or health care also are factors. Higher prevalence of chronic diseases further increases immune susceptibility and inflammation leading to greater COVID-19 morbidity and mortality (186). The need to accelerate efforts to promote health and to prevent, treat and reverse chronic diseases must be grounded in a commitment to greater equity for marginalized populations and reduction of exclusionary policies that block access to healthy behaviors. Leveraging emerging science, new application of research methods and technologies, and more appropriate evidence-grading systems to inform clinical care to deploy effective lifestyle medicine practices must become a significant emerging strategy to improve the lifespan and healthspan.

Finally, we believe that this Summit, its interdisciplinary framework, and recommendations are groundbreaking not only in the context of lifestyle medicine, but also potentially for application to other fields of medicine as well.

## Author Contributions

YV and MP wrote the manuscript. All authors participated in editing of the manuscript.

## Conflict of Interest

YV Co-founder of, and stakeholder in, Immunetrics, Inc. PV is the founder and interim CEO of Eodyne S L, which aims at bringing scientifically validated neurorehabilitation technologies to society. JJ Serves on the Scientific Advisory Board for WW International, Inc. and also serves on the Scientific Advisory Board for Spark360. TF is the Founder and Chief Scientific Officer of Generian Pharmaceuticals. DB has served as a paid consultant to Bayer, BeHealth Solutions, Cereve/Ebb Therapeutics, Emmi Solutions, National Cancer Institute, Pear Therapeutics, Philips Respironics, Sleep Number, and Weight Watchers International. He has served as a paid consultant for professional educational programs developed by the American Academy of Physician Assistants and CME Institute, and received payment for a professional education program sponsored by Eisai (content developed exclusively by Dr. Buysse). He is an author of the Pittsburgh Sleep Quality Index, Pittsburgh Sleep Quality Index Addendum for PTSD (PSQI-A), Brief Pittsburgh Sleep Quality Index (B-PSQI), Daytime Insomnia Symptoms Scale, Pittsburgh Sleep Diary, Insomnia Symptom Questionnaire, and RU_SATED (copyright held by University of Pittsburgh). These instruments have been licensed to commercial entities for fees. He is also co-author of the Consensus Sleep Diary (copyright held by Ryerson University), which is licensed to commercial entities for a fee. NB serves without compensation as president of the Physicians Committee for Responsible Medicine and Barnard Medical Center in Washington, DC, nonprofit organizations providing educational, research, and medical services related to nutrition. He writes books and articles and gives lectures related to nutrition and health and has received royalties and honoraria from these sources. The remaining authors declare that the research was conducted in the absence of any commercial or financial relationships that could be construed as a potential conflict of interest.
